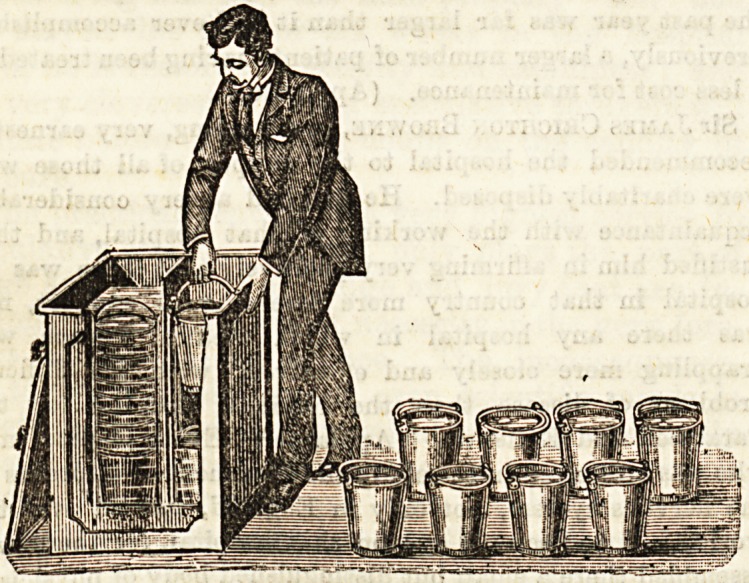# Bucket Fire Extinguisher

**Published:** 1892-06-04

**Authors:** 


### BUCKET FIRE EXTINGUISHER.

Fire buckets are essentials in all public and private institutions, and the space necessarily occupied by them, together with their liability to misuse and the oft required refilling has always been a disadvantage. Our illustration shows a means by which all these inconveniences are remedied. By this unique system of telescoping the buckets together beneath the water in a covered receptable, 10, 20, 30, or more buckets can be compactly stored in a very small space. The buckets, by the ends of their handles sliding in channels, are guided into and held in position ; they are instantly released by the action of raising their handles when lifting them out. So guided and retained it is impossible for a bucket to become in any way displaced or jammed, or to be lifted by the suction caused by the withdrawal of the bucket above it. By this simple arrangement the buckets on being withdrawn pick up their water and come out full, ready for immediate use, and can be taken in succession with the utmost rapidity. As the water and the buckets are in a closed vessel under seal (only to be broken in the event of fire) they are both perfectly protected from misuse. The cover of the tank being airtight, there is no evaporation of the water, nor that danger of freezing as with exposed buckets, and with the addition of a little disinfectant (such as Condy's fluid, permanganate of potash, &c.) the water may remain in the tank for quite twelve months without being changed or requiring any attention whatever. The Bucket Fire Extinguisher has no complicated parts, taps, or other fittings, liable to get out of order through long disuse. It is very compact, occupying so small a space that it can be placed almost anywhere, and may be made—where its normal appearance might be considered inadmissible—in the form of a pedestal, sideboard, settee, seat, &c., *en suite* with the furniture. It will be seen from the simplicity and compactness of the apparatus that its sphere of application is exceedingly wide and varied. The manufacturers of the Extinguisher are Messrs. Messer and Thorpe, Quality Court, Chancery Lane, and prices range from £7 7s. to £17.

## Figures and Tables

**Figure f1:**